# Ventilator-induced lung injury promotes inflammation within the pleural cavity

**DOI:** 10.1165/rcmb.2023-0332OC

**Published:** 2024-05-20

**Authors:** Rhianna F Baldi, Marissa W Koh, Chubicka Thomas, Tomasz Sabbat, Bincheng Wang, Stefania Tsatsari, Kieron Young, Alexander Wilson-Slomkowski, Sanooj Soni, Kieran P O’Dea, Brijesh V Patel, Masao Takata, Michael R Wilson

**Affiliations:** Division of Anaesthetics, Pain Medicine and Intensive Care, Department of Surgery & Cancer, Imperial College London, UK

**Keywords:** mechanical ventilation, acute respiratory distress syndrome, pleural cavity, mesothelium, animal model

## Abstract

Mechanical ventilation contributes to the morbidity and mortality of patients in Intensive Care, likely through the exacerbation and dissemination of inflammation. Despite its proximity to the lungs and exposure to physical forces, little attention has been paid to the potential of the pleural cavity as an inflammatory source during ventilation. Here we investigate the pleural cavity as a novel site of inflammation during ventilator-induced lung injury. Mice were subjected to low or high tidal volume ventilation strategies for up to 3 hours. High tidal volume ventilation significantly increased cytokine and total protein levels in bronchoalveolar and pleural lavage fluid. In contrast acid aspiration, explored as an alternative model of injury, only promoted intra-alveolar inflammation with no effect on the pleural space. Resident pleural macrophages demonstrated enhanced activation following injurious ventilation, including upregulated ICAM-1 and interleukin-1β expression, and release of extracellular vesicles. In vivo ventilation and in vitro stretch of pleural mesothelial cells promoted ATP secretion, while purinergic receptor inhibition substantially attenuated extracellular vesicles and cytokine levels in the pleural space. Finally, labelled protein rapidly translocated from the pleural cavity into the circulation during high tidal volume ventilation, to a significantly greater extent than protein translocation from the alveolar space. Overall we conclude that injurious ventilation induces pleural cavity inflammation mediated via purinergic pathway signaling, and likely enhances dissemination of mediators into the vasculature. This previously unidentified consequence of mechanical ventilation potentially implicates the pleural space as a focus of research and novel avenue for intervention in critical care.

## Introduction

Mechanical ventilation is a crucial component of supportive treatment in the Intensive Care Unit (ICU), both for patients suffering from respiratory failure and those undergoing major surgical procedures. It is however widely accepted that mechanical ventilation invokes non-physiological forces within the lung which can worsen outcome, a phenomenon termed ventilator-induced lung injury (VILI). The ‘biotrauma’ hypothesis of VILI proposes that ventilation exacerbates inflammation, and that this is causally linked to permeability and physiological dysfunction of the lungs. Following systemic dissemination, this inflammation is further suggested to contribute to the multiple system organ failure commonly observed in acute respiratory distress syndrome (ARDS) patients (1, 2).

While the inflammatory consequences of mechanical ventilation within the lungs themselves have been much studied, there has been almost no consideration given to the pleural cavity, despite its intimate relationship with the lung parenchyma. Pleural effusions are a very common finding in the ICU occurring in up to 80% of ventilated ARDS patients, and are associated with longer duration of ventilation and ICU stay, although the reasons are uncertain (3). Pleural fluid drainage has been shown to improve oxygenation and possibly respiratory mechanics, although whether physical constraints of effusions (e.g. compression of respiratory structures) significantly impact clinical outcomes is questionable (4). It has been shown in septic patients that pleural fluid samples can contain high levels of inflammatory mediators (5), and while the source of such mediators is unknown, the pleural cavity contains populations of cavity specific macrophages (6) and other leukocytes which could potentially be a source of locally produced cytokines. Moreover, mesothelial cells lining the pleural cavity are exposed to substantial mechanical forces during breathing. Pleural mesothelia undergo significant cyclic stretching during lung expansion (7, 8), while movement of the visceral and parietal pleura relative to each other imposes substantial shear stress (9). Cultured pleural mesothelial cells are capable of releasing a wide range of mediators following inflammatory stimuli (10). Furthermore, both mechanical stretching and shear stress induce the release of endothelin-1 from mesothelial cells in vitro (11).

Within this study we therefore hypothesised that high tidal volume mechanical ventilation induces inflammation within the pleural cavity as a result of pleural cell activation. Our findings indicate that VILI (perhaps uniquely) both upregulates pleural cavity cytokines and enhances dissemination of proteins into the circulation, potentially implicating the pleural space as an important locus of inflammation and therapeutic target within critically ill patients. Some of the results of these studies have been previously reported in the form of an abstract (12).

## Methods

Detailed methods are provided in an online supplement.

### Injury models

Animal experiments were performed under the Animals (Scientific Procedures) Act 1986, UK, using male C57BL/6 mice aged 8-12 weeks. The in vivo VILI model has been described previously (13, 14) - see online supplement for details. Anesthetised mice were ventilated using either an injurious high tidal volume (V_T_) strategy (V_T_ 32-36ml/kg), or protective low V_T_ (7-8ml/kg, with recruitment manoeuvres every 30 minutes). Mice were ventilated for pre-determined periods up to 3 hours, or until a mortality surrogate was reached. In inhibition studies, 50μl saline or PPADS (purinergic receptor inhibitor) were delivered intrapleurally before ventilation protocols.

As a comparison to the VILI model, experiments using an acute acid aspiration model were carried out (15). Mice were anesthetised, intratracheally instilled with saline or 0.15M HCl (50μl) and ventilated with low V_T_ (including recruitment manoeuvres) for 3 hours.

After termination plasma and bronchoalveolar lavage fluid (BALF) samples were collected. Pleural lavage fluid (PLF) was recovered by piercing the parietal pleura, instilling 400μl saline and withdrawing as much as possible from the cavity. Cytokine concentrations were determined by ELISA, while ATP levels were determined using ATPlite kit. Flow cytometry was used to assess activation of pleural cavity cells via surface and intracellular markers, as well as production of extracellular vesicles (EV) (16).

### Protein movement studies

To determine blood-to-pleural cavity and blood-to-alveolar space permeability during VILI, 50μl Alexa-Fluor 594-conjugated albumin was injected intravenously. After 3 hours low or high V_T_ ventilation, plasma, BALF and PLF were collected and fluorescence evaluated. Plasma fluorescence was determined after 100-fold dilution while BALF and PLF samples were measured undiluted. Dye movement was evaluated as ratio of fluorescence units in BALF or PLF versus plasma.

To determine pleural cavity-to-blood or alveolar space-to-blood movement of protein, 50μl AF594-albumin was instilled into the pleural cavity or alveolar space, followed by ventilation for 3 hours low or high V_T_ ventilation. High V_T_ settings were matched based on plateau pressure to invoke similar degrees of lung strain (34.3±2.27 vs 33.3±1.47 cmH_2_O for intrapleural vs intratracheal dye respectively). Serial plasma samples from intrapleurally- and intratracheally-dosed mice were collected at intervals throughout ventilation and fluorescence determined. Dye movement was evaluated as ratio of fluorescence units in undiluted plasma versus initial instillate (following 100-fold dilution).

### In vitro studies

Rat pleural mesothelial cells (4/4 RM-4) were seeded onto collagen-coated flexible bottom plates and exposed to 20% cyclic stretch (or held static) for 15 minutes or 4 hours, using a Flexcell FX-6000 Tension system. Following 15 minutes experiments, supernatants were collected, ARL67156 (ecto-ATPase inhibitor) was added, and ATP levels determined. Following 4 hours experiments, supernatants were recovered and EV numbers determined by flow cytometry (16).

### Statistics

Data were evaluated for distribution by Shapiro-Wilk test of residuals. Normally distributed data were analysed by t-test, one-way ANOVA with Sidak’s test or two-way ANOVA with Tukey’s multiple comparisons test. Non-normally distributed data were analysed by Mann-Whitney test or Kruskal-Wallis followed by Dunn’s test.

## Results

**Figure 1:**
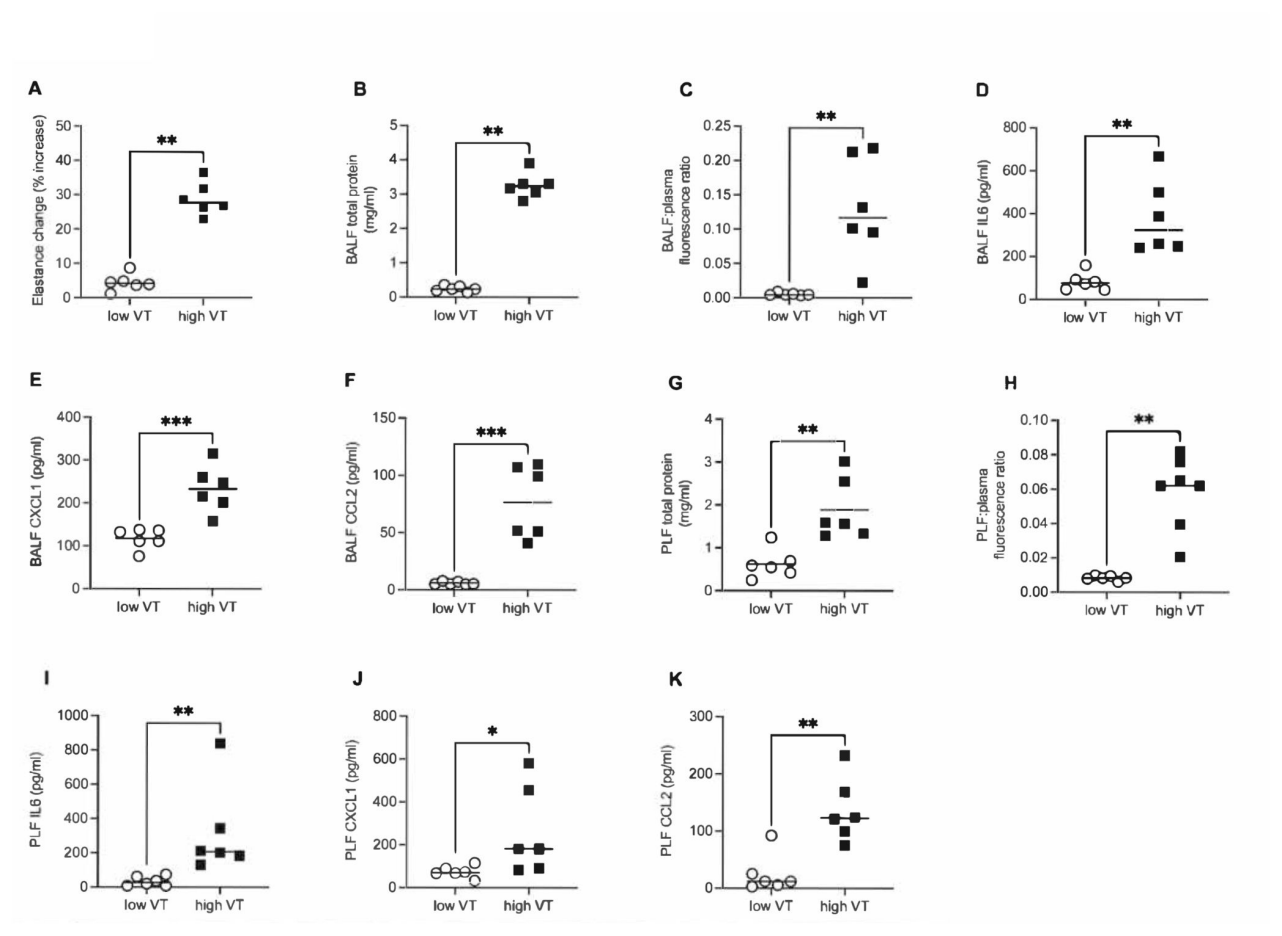
Permeability and inflammation in alveolar and pleural compartments during VILI. Samples were taken at either 3 hours of ventilation, or when injury had reached a point to attain the mortality surrogate if this occurred first (only with high tidal volume (V_T_) mice). Elastance change (A) was calculated as percentage increase compared to the value at the start of either high or low V_T_ ventilation. Concentration of total protein in bronchoalveolar lavage fluid (BALF) (B), and the ratio of AlexaFluor 594-albumin present in BALF to that in plasma (C) were determined. Markers of inflammation IL-6 (D), CXCL1 (E) and CCL2 (F) were measured in BALF by ELISA. Similarly, total protein in pleural lavage fluid (PLF) (G), ratio of AlexaFluor 594-albumin present in PLF to that in plasma (H) and pleural fluid IL-6 (I), CXCL1 (J) and CCL2 (K) were evaluated. Data in panels A-D and H-K were non-normally distributed and analysed by Mann-Whitney test, while data in panels E-G were normally distributed and analysed by student’s t-test. Data are displayed as individual data points with solid line indicating either mean or median value for normal or non-normal data respectively. N=6-7 for each dataset. *p<0.05, **p<0.01, ***p<0.001.

The physiological characteristics of the VILI model are shown in Supplemental Figure E1. Consistent with previous studies (14, 17), in this ‘one-hit’ model of high V_T_ ventilation, respiratory mechanics were initially stable until ~90 minutes, following which there was a relatively rapid deterioration. In contrast low V_T_ ventilation with recruitment manoeuvres induced no impairment in respiratory mechanics, and all animals survived until the predetermined end-point. As expected, 3 hours high V_T_ induced substantial increases in respiratory system elastance (Fig 1A), BALF total protein (Fig 1B), and fluorescent marker translocation from blood to BALF (reflecting enhanced barrier permeability) (Fig 1C). In addition, high V_T_ promoted significant increases in BALF cytokines (Fig 1D-F).

On evaluation of pleural fluid samples (PLF) we similarly observed that total protein levels were raised following 3 hours of high V_T_, associated with increased permeability of the pleural barriers (Figure 1G/H). Moreover, we also observed substantial increases in inflammatory cytokines IL-6, CXCL1 and CCL2 in the pleural space (Fig 1I-K). Of note, the levels of PLF cytokines were similar in magnitude to those found in plasma (IL-6; PLF 318±264 pg/ml vs plasma 604±165 pg/ml: CXCL1; PLF 261±206 pg/ml vs plasma 481±272 pg/ml: CCL2; PLF 137±56 pg/ml vs plasma 125±48 pg/ml).

**Figure 2:**
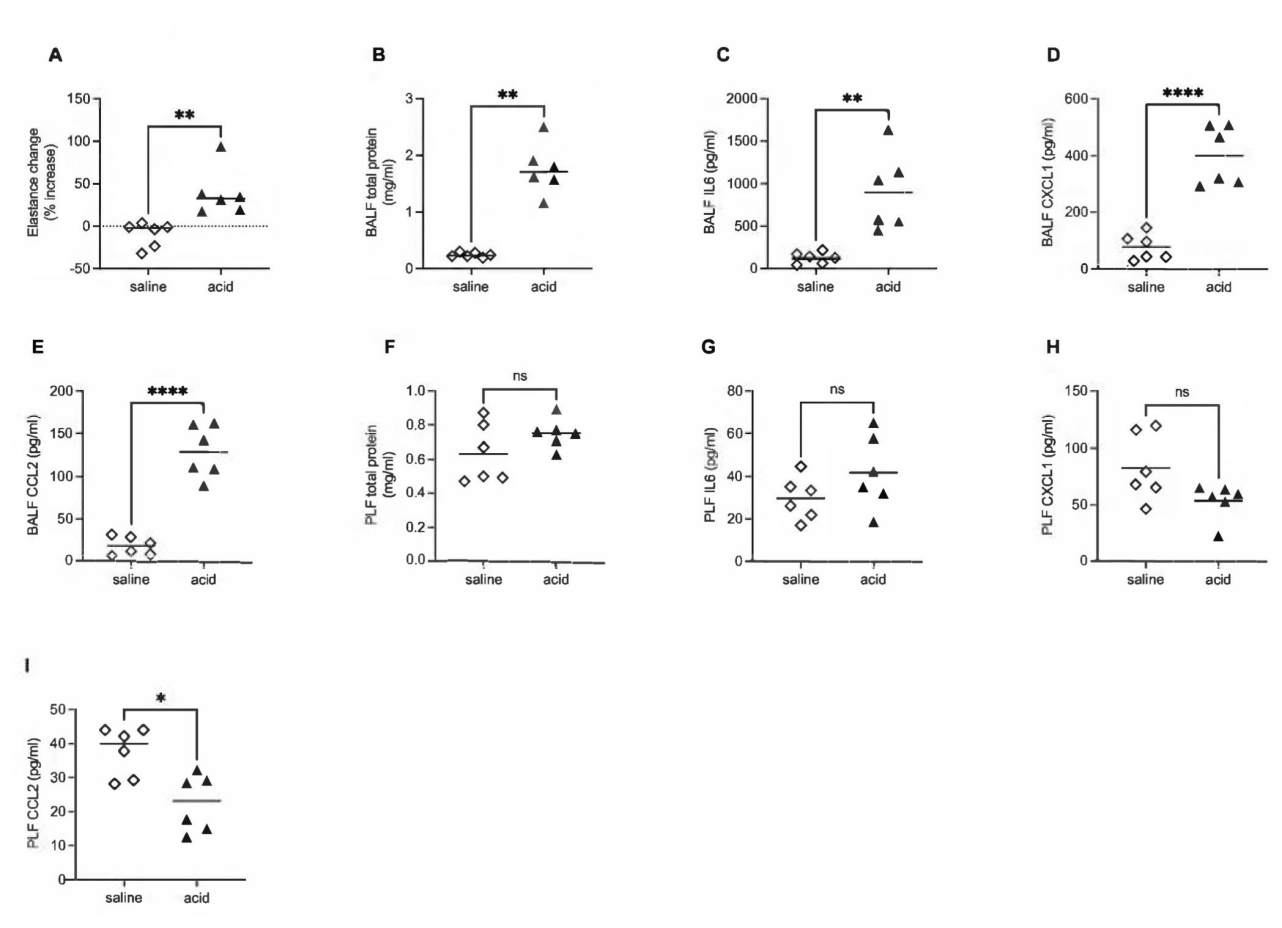
Permeability and inflammation in alveolar and pleural compartments in acid aspiration model. Samples were taken 3 hours after either saline or HCl instillation. Elastance change (A) was calculated as percentage increase compared to the value immediately after instillation of either saline or HCl. Concentration of BALF total protein (B), IL-6 (C), CXCL1 (D), CCL2 (E), and pleural lavage fluid (PLF) total protein (F), IL-6 (G), CXCL1 (H) and CCL2 (I) were determined. Data in panel A, B and I were non-normally distributed and analysed by Mann-Whitney test, while data in panels C-H were normally distributed and analysed by student’s t-test. Data are displayed as individual data points with solid line indicating either mean or median value for normal or non-normal data respectively. N=6 for each dataset. *p<0.05, **p<0.01, ****p<0.0001.

To explore whether this phenomenon was specific to VILI, we carried out similar measurements in a model of acute acid aspiration lung injury. As with the VILI model, acid aspiration induced significant increases in respiratory elastance and resistance over 3 hours (Supplemental Figure E2, Fig 2A), along with increases in BALF total protein, IL-6, CXCL1 and CCL2 (Fig 2B-E). Notably, elastance increase and BALF cytokine concentrations were higher following acid aspiration compared to VILI. However, in complete contrast to the VILI model, acid aspiration did not lead to increases in total protein or any cytokines measured within the pleural space (Fig 2F-I).

**Figure 3:**
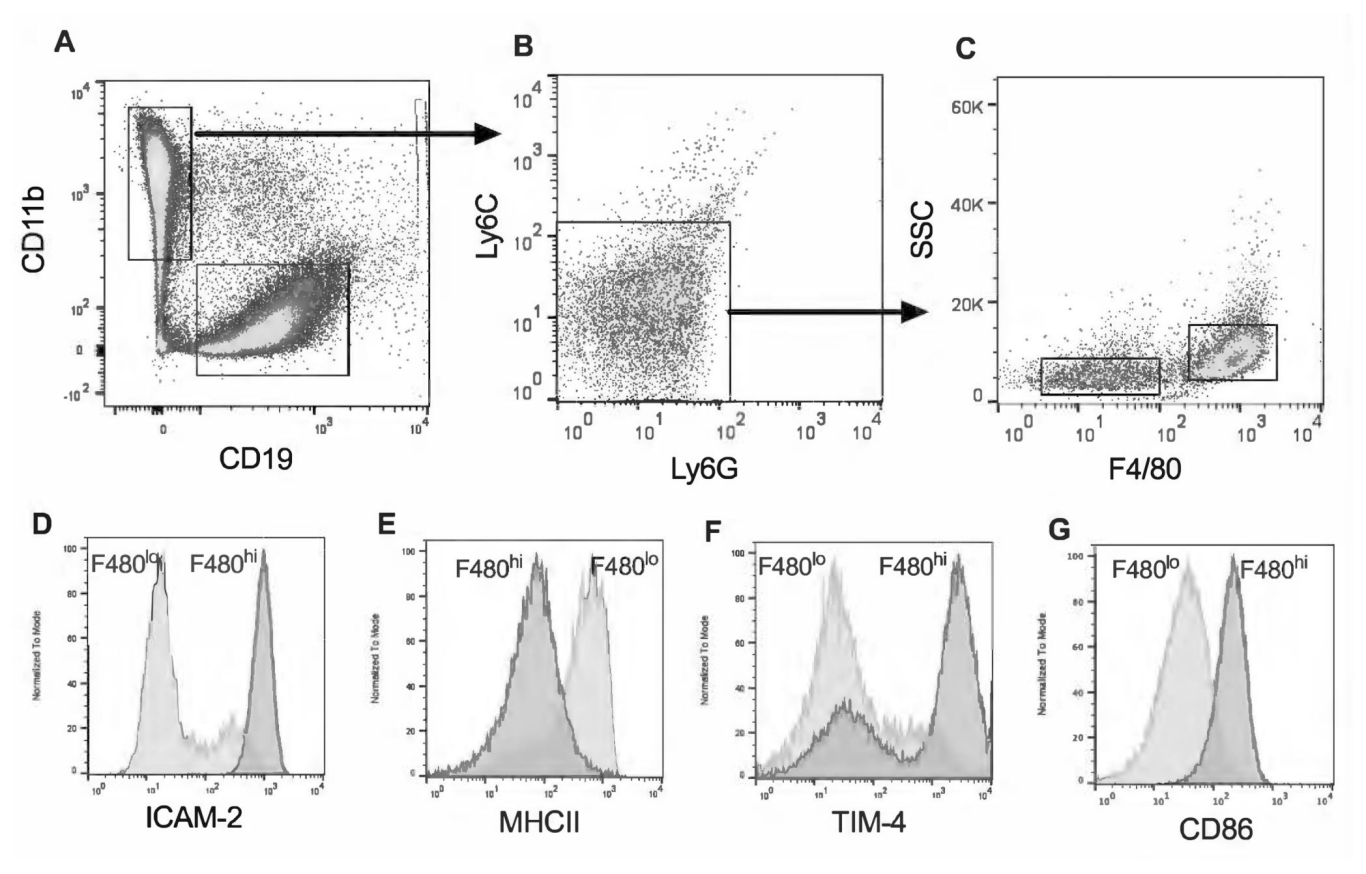
Identification of cells recovered from pleural cavity lavage. Cells were first distinguished based on expression of CD11b and CD19 (A). CD11b^+^ cells were then confirmed as being negative for the circulating monocyte marker Ly6C and the neutrophil marker Ly6G (panel B). CD11b^+^ cells could be identified as 2 distinct populations based on expression of F480 as well as side scatter (SSC) characteristics (C) and expression of ICAM2 (D), MHCII (E), TIM-4 (F) and CD86 (G).

**Figure 4:**
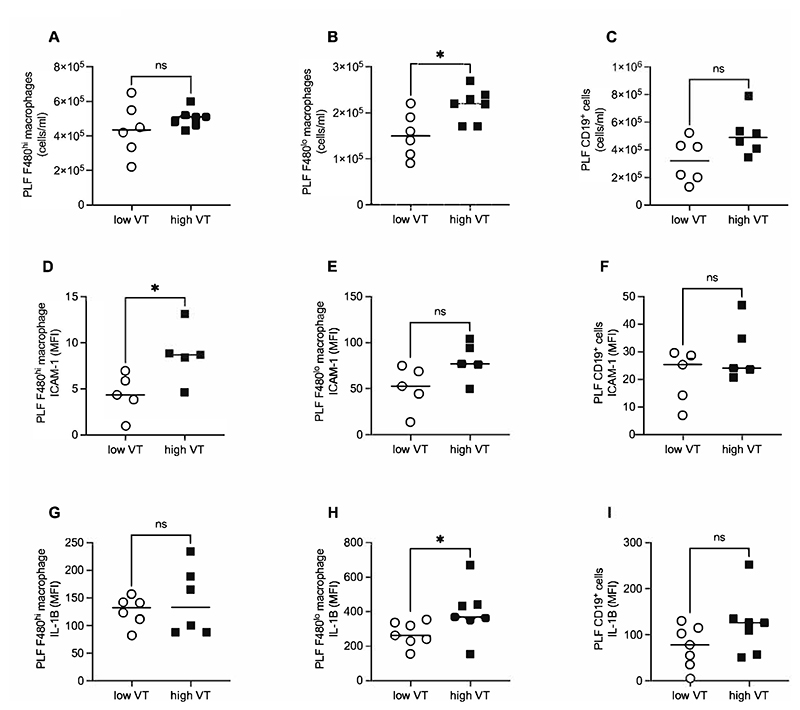
Cell quantification and activation in pleural lavage fluid (PLF) samples by flow cytometry. Numbers of F480^hi^ macrophages (A), F480^lo^ macrophages (B), and CD19^+^ cells (C) were determined following 3 hours of high or low V_T_ ventilation. Additionally, surface ICAM-1 expression on F480^hi^ macrophages (D), F480^lo^ macrophages (E), and CD19^+^ cells (F), and whole cell IL-1β expression in F480^hi^ macrophages (G), F480^lo^ macrophages (H), and CD19^+^ cells (I) were evaluated. Data were non-normally distributed and analysed by Mann-Whitney test. Data are displayed as individual data points with solid line indicating median value. N=5-7 for each dataset. *p<0.05.

As resident macrophages have previously been implicated in inflammatory responses within the pleural cavity (18), we focussed our investigations on whether these may be responsible for inflammation during VILI, with B-cell responses explored as a comparator. Pleural lavage fluid cells were first identified based on expression of CD11b and CD19, the majority of PLF cells falling into either CD11b^+^CD19^-^ or CD11b^-^CD19^+^ populations (Figure 3A). CD19^+^ cells were assumed to be populations of B-cells (19), while CD11b^+^CD19^-^ cells were confirmed as macrophages based on absence of circulating monocyte (Ly6C) and neutrophil (Ly6G) markers (Fig 3B). Consistent with previous findings macrophages existed as 2 distinct populations, a major population of F480^hi^ cells showing higher granularity (Fig 3C), higher expression of ICAM2 (Fig 3D), TIM-4 (Fig 3F) and CD86 (Fig 3G) but lower expression of MHCII (Fig 3E) compared to F480^lo^ macrophages (19–21). After 3 hours of high V_T_ ventilation the numerically dominant cell populations in the pleural cavity (F480^hi^ macrophages and CD19^+^ cells) were unchanged in number, though there was a small but significant increase in the number of F480^lo^ macrophages (Fig 4A-C). Numbers of neutrophils and monocytes remained negligible (not shown). To clarify whether leukocytes in the pleural cavity were activated during high V_T_ ventilation, and thus potentially responsible for inflammatory mediator production, we evaluated surface ICAM-1 and whole cell IL-1β expression by flow cytometry. ICAM-1 expression was significantly increased on F480^hi^ macrophages and showed a close to significant increase on F480^lo^ macrophages (p=0.056), but not on CD19^+^ B-cells (Fig 4D-F). Cellular IL-1β levels were significantly increased in F480^lo^ macrophages (Fig 4G-I), indicating that macrophages specifically showed signs of inflammatory activation during VILI.

**Figure 5:**
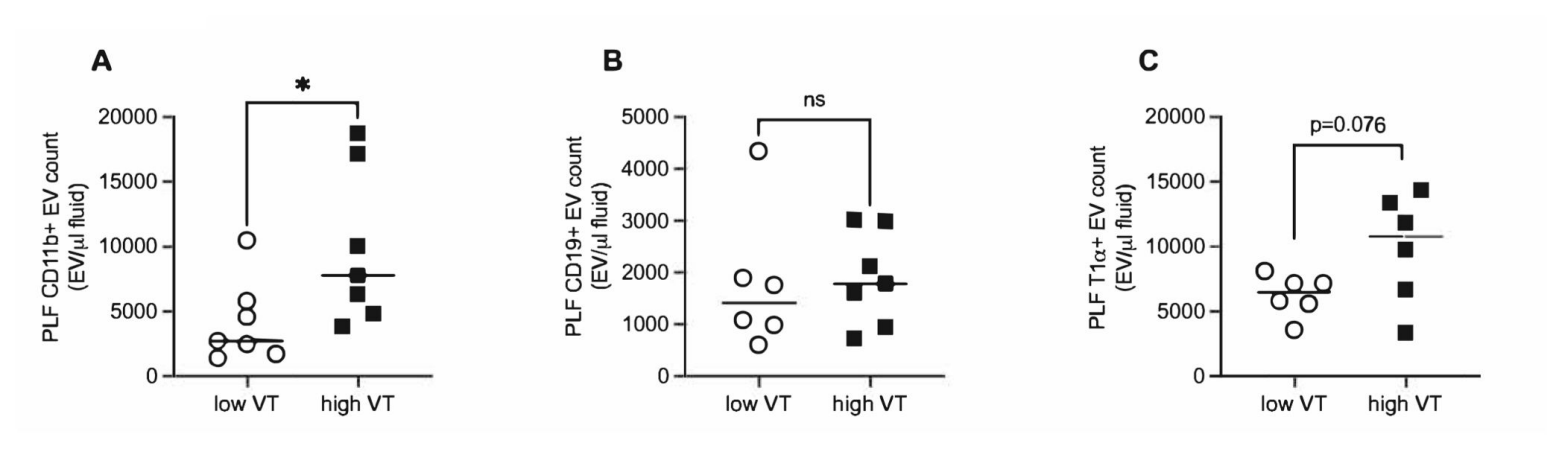
Extracellular vesicle identification and quantification in pleural lavage fluid (PLF) samples by flow cytometry. Extracellular vesicles expressing surface markers for CD11b (A), CD19 (B) and T1α (C) were quantified after 1 hour of high or low V_T_ ventilation. Data were non-normally distributed and analysed by Mann-Whitney test. Data are displayed as individual data points with solid line indicating median value. N=6-7 for each dataset. *p<0.05.

To further explore the nature of inflammatory responses we determined the numbers of extracellular vesicles (EV) released from the different resident cell types identified previously, as markers of cellular activation. These measurements were carried out at an earlier time point of ventilation (1 hour) to minimise possible confounding effects of EV ingress from outside the pleural space or uptake by cells. The data demonstrate a significant increase in the number of CD11b^+^ EVs in the pleural fluid after just 1 hour of high V_T_ (Fig 5A). MHCII^+^ and ICAM2^+^ EVs showed a numerical, but not statistically significant increase (data not shown), while CD19^+^ EVs were unchanged (Fig 5B). Interestingly, there was a close to significant (p=0.076) increase in T1α^+^ EVs (Fig 5C) suggesting the possibility of mesothelial cell activation. To confirm that EV data represents local inflammatory cell activation rather than infiltration from elsewhere (e.g. the alveolar space, as T1α is also present on alveolar epithelial cells), EVs expressing either CD11b or CD11c (alveolar macrophage marker) were determined in pleural fluid and BAL fluid following 1 hour high or low V_T_. Consistent with the concept of a local EV source, CD11c^+^ EV numbers only increased in BAL fluid following VILI, while CD11b^+^ EVs only increased in pleural lavage fluid (Supplemental Fig E3). Thus the data indicate little (if any) direct movement of mediators between the alveolar and pleural space.

**Figure 6:**
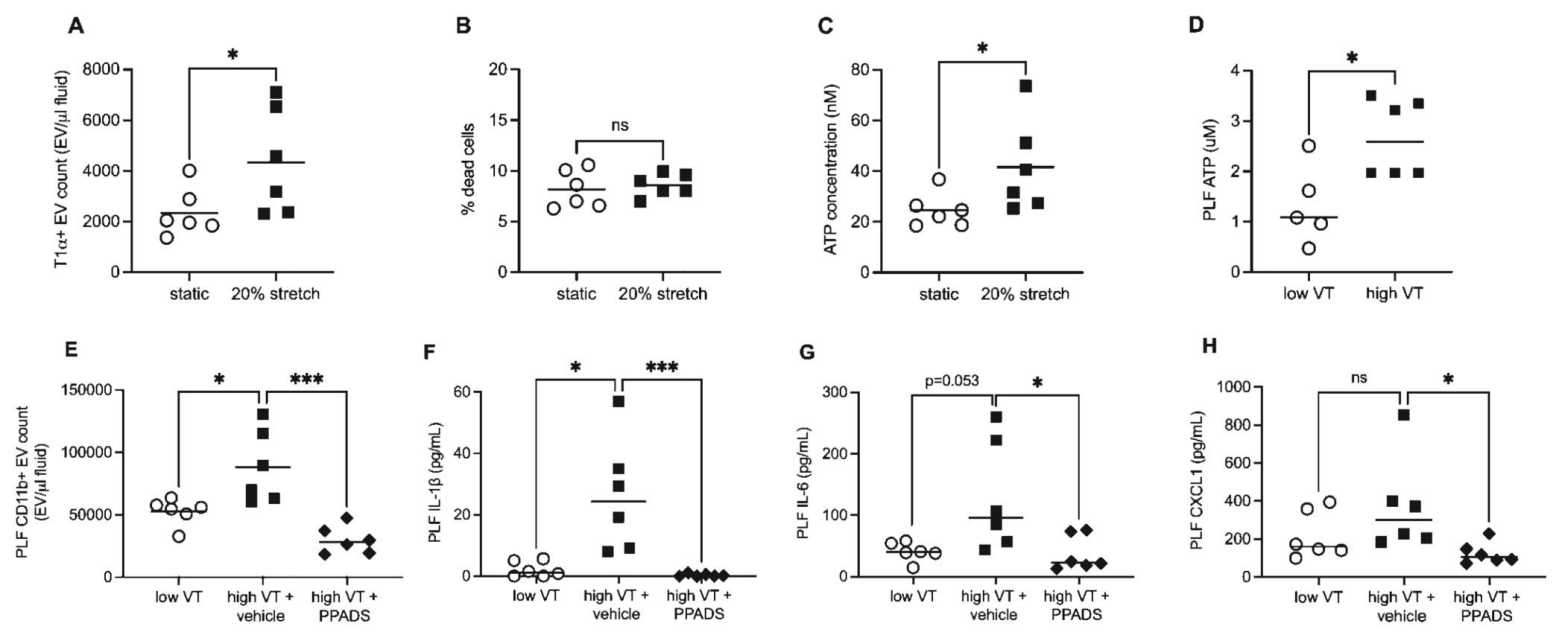
Pleural cavity purinergic receptor involvement in VILI. Pleural mesothelial cells (4/4 RM-4) were exposed to 20% cyclic stretch or held static for 4 hours for measurement of T1α^+^ extracellular vesicles (A). Presence of lactate dehydrogenase (LDH) was evaluated in terms of % dead cells (% signal following cell lysis) to clarify whether cell stretching was associated with cell death (B). (C) pleural mesothelial cells were exposed to 20% cyclic stretch or held static for 15 minutes for measurement of ATP secretion. Subsequently, ATP concentration was evaluated in pleural lavage fluid (PLF) samples from mice ventilated with high or low V_T_ for 1 hour (D). In separate experiments, mice were dosed intrapleurally with 50μl saline or 50mM PPADS before ventilation with high or low V_T_ for 1 hour. PLF samples were evaluated for CD11b^+^ EVs by flow cytometry (E), and soluble IL-1β (F), IL-6 (G) and CXCL1 (H). (Note that data in 6E were obtained with a more sensitive flow cytometer than data in Fig 5, hence differences in absolute CD11b^+^ EV counts between figures). Data in panels A, B, C and E were normally distributed and analysed by t-test or one-way ANOVA with Sidak’s multiple comparisons test as appropriate. Data in panels D, F, G and H were non-normally distributed and analysed by Mann-Whitney test or Kruskal-Wallis with Dunn’s multiple comparisons test. Data are displayed as individual data points with solid line indicating mean or median value. N=5-6 for each dataset. *p<0.05, ***p<0.001.

Given the above findings, we postulated a mechanistic paradigm whereby exposure of mesothelial cells to stretch induces release of factors which subsequently promote local macrophage responses. Initially, in vitro studies were carried out which showed that mesothelial cells exposed to stretch for 4 hours released T1α^+^ EVs, with no significant increase in cell death based on LDH assay (Fig 6A/B). As a known DAMP, we determined ATP secretion from mesothelial cells and found a significant increase after just 15 minutes of stretch (Fig 6C). Subsequently, we explored whether ATP released from stretched mesothelial cells may be responsible for pleural macrophage activation in vivo. Firstly, we determined ATP concentration within the pleural cavity, which was significantly increased after 1 hour of high V_T_ (Fig 6D). We then carried out experiments utilising the non-specific purinergic receptor blocker PPADS, which demonstrated that both CD11b^+^ EV production and pro-inflammatory cytokine upregulation were attenuated by purinergic receptor inhibition (Fig 6E-H).

**Figure 7:**
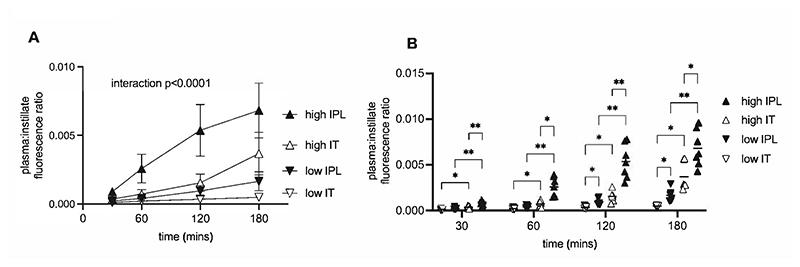
Translocation of labelled protein from pleural cavity or alveolar space to circulation. Time course of appearance of AlexaFluor 594-albumin within plasma, expressed as a ratio of plasma fluorescence to that of the original instillate during 3 hours of high or low V_T_ ventilation. Low IT = low V_T_, intratracheal dye; Low IPL = low V_T_, intrapleural dye; High IT = high V_T_, intratracheal dye; High IPL = high V_T_, intrapleural dye. Data were normally distributed and were analysed by 2-way ANOVA followed by Tukey’s multiple comparisons test. Panel A shows time course of changes within different groups, with data displayed as mean ± SD. Time x group interaction showed significance at p<0.0001. Panel B shows comparisons at each time point, displayed as individual data points with differences between each group at a given time shown as *p<0.05, **p<0.01. N=6-7 for each dataset at each time point.

Finally, having demonstrated the initiation of an inflammatory response within the pleural cavity during high V_T_ ventilation, we considered whether this remained a localised response or whether there was the potential for inflammatory mediators to disseminate. We therefore determined the movement of fluorescence-labelled albumin out of the pleural and alveolar spaces during 3 hours ventilation. The immediate impact of bolus administration on respiratory mechanics meant that despite matched plateau pressure, tidal volume was slightly (though not significantly) lower in high V_T_ mice following intratracheal administration of protein compared to intrapleural administration (28.3±3.04 vs 31.2±2.64 ml/kg). However, the increase in elastance over the time course was similar between intratracheally and intrapleurally-dosed mice ventilated with high V_T_ (10.3±14.0 vs 5.59±6.19 % increase respectively) indicating that similar degrees of VILI were induced. Following intrapleural administration, there was a gradual appearance of dye within plasma over the course of 3 hours low V_T_ ventilation, which was dramatically enhanced at all time points by high V_T_ ventilation (Fig 7). Following intratracheal administration, high V_T_ also increased dye appearance in plasma compared to low V_T_. However, amounts of dye translocating to plasma were significantly lower following intratracheal administration compared to intrapleural administration, regardless of ventilation strategy (Fig 7). Overall these data demonstrate that high V_T_ ventilation promotes movement of protein from the pleural space to the circulation, and does so to a greater extent / more rapidly than occurs from the alveolar space.

## Discussion

While mechanical ventilation is a life-saving component of ICU treatment, the potential for negative consequences is well-known, including the suggestion that ventilation exacerbates the development of multiple system organ failure among ARDS patients. A number of possible explanations for this have been proposed (22), but most focus has been attached to the inflammatory response. Both local intra-pulmonary and circulating cytokines have been linked to VILI in preclinical models, and associated with increased mortality in ARDS patients, leading to the concept that mechanical ventilation promotes organ injury via the systemic propagation of inflammation (2). Explanations for this dissemination of mediators involve either passive leak from alveolus to blood, or ventilation directly impacting on endothelial cells and/or leukocytes within the pulmonary vasculature (23, 24). Perhaps surprisingly, there has been no consideration of a possible involvement of the pleural cavity, even though it is exposed to substantial physical forces during lung expansion. In this study we show for the first time that injurious mechanical ventilation provokes inflammation within the pleural space, which may contribute to the inflammatory load of patients.

In our initial experiments, we determined the levels of inflammatory cytokines in BAL and pleural fluid of ventilated animals. As expected, inflammatory mediators within the alveolar space were increased with high V_T_, but we also demonstrated heightened inflammation within the pleural cavity. Importantly, the levels of cytokines in pleural lavage fluid following VILI were comparable in magnitude to the concentration in plasma. The mouse pleural cavity comprises a single compartment surrounding both lungs, containing a volume of ~20μl fluid (25). Here, we utilised a lavage recovery procedure involving the instillation of 400μl saline, allowing washout of the cavity surrounding both lungs but diluting pleural lining fluid approximately 20-fold. Thus, the cytokine levels in pleural fluid before dilution would likely be substantially higher than observed in plasma, consistent with local production rather than movement from the circulation (26). Total protein levels in pleural lavage fluid were significantly increased following high V_T_ ventilation which, given that plasma protein levels were unchanged between low and high V_T_ (27.7±1.88 vs 28.2±3.57 mg/ml), is consistent with development of an exudative effusion (27) during VILI. It should be pointed out that in these initial experiments, only 1/6 mice survived the full 3-hour high V_T_ protocol, with others meeting the mortality surrogate earlier. High V_T_ animals were on average therefore ventilated for less time than other groups. This is however very unlikely to have any material impact on our conclusions as analysis (not shown) found that all mediators / markers of permeability tended to increase with longer high V_T_ ventilation time. Nevertheless, for subsequent experiments ventilation settings were adjusted slightly to ensure that all high V_T_ animals survived the entire period.

Crucially, within an acid aspiration model of lung injury which induced similar alveolar-epithelial permeability, and even greater increases in BALF cytokines than the VILI model, we found no increases in pleural fluid protein or cytokine concentrations. Thus, the development of pleural cavity inflammation seems to be a unique response to VILI compared to other etiologies of acute lung injury, at least within this early phase.

The major leukocyte types identified within the pleural cavity, which we hypothesised may be responsible for the aforementioned cytokine secretion, included CD19^+^ B-cells and CD11b^+^ macrophages, the latter of which existed in two distinct populations based on relative expression of F480, MHCII, ICAM2, TIM-4 and CD86 (19–21). The numerically dominant F480^hi^ MHCII^lo^ population is considered to be a self-proliferating population derived from embryonic precursors, whereas the F480^lo^ MHCII^hi^ population is derived from circulating monocytes (20). While there was no change in the numbers of F480^hi^ macrophages, the number of F480^lo^ macrophages did increase significantly with high V_T_ ventilation. It is unclear whether this reflects recruitment of cells or altered efficiency of recovery.

As confirmation that resident pleural cavity cells were indeed directly activated by VILI, we demonstrated increased expression of ICAM-1 and IL-1β on/in macrophages, but not B-cells, by flow cytometry. ICAM-1 was used as we have previously demonstrated this to be a robust marker of alveolar macrophage activation during VILI (14). While ICAM-1 expression was only significantly increased on F480^hi^ cells, there was a numerical increase on F480^lo^ macrophages. These had substantially higher basal expression levels than F480^hi^ cells, which may have made detection of a signal more difficult. ICAM-1 has traditionally been explored for a role in leukocyte migration, but recently has been shown to also play important roles in activation of tissue resident leukocytes (28).

To gain further insight into the inflammatory responses during ventilation, we investigated extracellular vesicle (EV) production from resident cells. These measurements were carried out at an earlier time point of ventilation (1 hour) both to minimise the possibility that these EV may be translocating in from elsewhere, and to minimise loss of EVs as a result of uptake by cells (29). Of note, our protocols are designed to recover a population consisting primarily of microvesicles (16), but we cannot rule out the presence of other EVs including large exosomes (although this distinction is not particularly relevant here given that we are using these measurements purely to gain information regarding the cell types activated, rather than invoking them as playing a pathophysiological role). Consistent with our previous observation of macrophage activation, we found significant upregulation of CD11b^+^ EVs, but not CD19^+^ EVs. Intriguingly, there were indications in our study (though not significant) of upregulated T1α^+^ EVs in the pleural space. T1α (podoplanin) is known to be abundantly expressed on the surface of pleural mesothelium (30), suggesting a direct response of the mesothelial cells to VILI. We subsequently demonstrated that pleural mesothelial cells exposed to stretch in culture released increased numbers of T1α^+^ EVs, confirming that these cells respond to cyclic stretch. It is theoretically possible that the T1α^+^ EVs observed in vivo could have moved from the alveolar space, as T1α is also present on the surface of alveolar epithelial cells. However, we believe this is very unlikely as alveolar macrophage-derived (CD11c^+^) EVs were increased in BAL fluid but not pleural lavage fluid following VILI. These findings further support the conclusion that local macrophages were activated and that our observations were not due to spill-over of mediators from the alveolar to the pleural space.

With regards to the precise mechanisms involved, we postulated a paradigm whereby deformation of visceral and /or parietal pleural mesothelial cells during high V_T_ ventilation leads to release of signals which promote macrophage activation and consequent secretion of inflammatory cytokines into the pleural space. ATP is known to be rapidly released from a wide variety of cells exposed to mechanical stimulation (31, 32) and extracellular ATP is an important damage-associated molecular pattern (DAMP) promoting inflammasome activation in macrophages. We therefore considered this to be a strong candidate as a signal that may be released by pleural mesothelium during VILI. Here we showed for the first time that pleural mesothelial cells exposed to cyclic stretch in vitro release ATP, and that in vivo VILI led to upregulated extracellular ATP in the pleural cavity. Moreover, use of the non-specific purinergic receptor inhibitor PPADS promoted almost complete abrogation of CD11b^+^ (pleural macrophage) EVs release and soluble IL-1β, IL-6 and CXCL1. While the precise purinergic receptor remains uncertain, these data strongly support the hypothesis that extracellular ATP (or potentially other nucleotide DAMPs) released from stretched pleural mesothelial cells promotes local macrophage-driven inflammation. We cannot exclude that mesothelial cells might also directly contribute to cytokine upregulation, and indeed they have been reported to be responsible for mediator production during pleural disease (33), and recently during COVID-19 related fibrotic damage (34). However our findings would suggest that in the early phase of VILI at least, this is likely to be a minor contribution compared to that of macrophages.

The pathophysiological consequences of our finding of ventilator-induced pleural inflammation remain unclear. A thorough exploration of this lies beyond the scope of this study, although we would speculate that it may have more impact on extrapulmonary consequences of ventilation rather than a compulsory role in lung dysfunction per se (at least in the very acute phase explored here). We show that high V_T_ ventilation, as well as inducing pleural cavity inflammation, also dramatically and rapidly enhances the egress of protein from the cavity into the circulation. Interestingly, movement from the pleural cavity seems to be of greater magnitude and/or rapidity than movement of protein from the alveolar space. The kinetics of dye appearance in plasma indicate that intrapleural protein seemed to translocate in a consistent manner over time during high V_T_ ventilation, whereas intra-alveolar protein translocation remained relatively limited until displaying a more rapid increase towards the end of experiments. We would speculate that this may reflect different mechanisms of movement from the two compartments. More rapid translocation from the pleural cavity may reflect either greater permeability of the mesothelium-to-circulation barriers, or alterations in lymphatic transport. This latter is a major mechanism of protein movement out of the pleural space (35), and crucially, lung lymph flow has been shown to be increased with greater tidal volume ventilation (36). (To note, increased respiratory rate has also been reported to promote epithelial cell permeability (37) and lung lymph flow (36), although in the current study our VILI model involved a reduction of respiratory rate, so increased movement of mediators would be purely related to increased V_T_). In contrast, the ‘late’ increase in dye translocation from the alveolar space likely reflects the development of epithelial barrier breakdown. Thus the data would indicate that injurious mechanical ventilation increases inflammatory cytokine levels within both the pleural cavity and alveolar space, but that egress from the pleural cavity into the circulation is likely to occur earlier, in the absence of overt lung injury.

The biotrauma hypothesis of ventilator-induced lung injury essentially proposes that ventilation enhances extra-pulmonary inflammation, organ injury and mortality via ‘spill-over’ of cytokines from within the lungs or pulmonary vasculature (23). Here we have demonstrated for the first time that a possible alternative mechanism exists, whereby VILI uniquely promotes both pleural cavity inflammation and the likely rapid dissemination of mediators from the pleural space into the vasculature. While the precise relevance of this still remains to be proven (as indeed, does any paradigm linking ventilation, inflammation and organ failure) this novel mechanism potentially opens new therapeutic avenues, not least because delivery of agents into the pleural space may be both more direct and less hampered by physical considerations (e.g. edema, altered regional perfusion) than other approaches.

## Supplementary Material

Fig. E1

Fig. E2

Fig. E3

Fig. E4

Fig. E5

Supplement

## References

[1] Ranieri VM, Suter PM, Tortorella C, De Tullio R, Dayer JM, Brienza A, Bruno F, Slutsky AS (1999). Effect of mechanical ventilation on inflammatory mediators in patients with acute respiratory distress syndrome: a randomized controlled trial. JAMA.

[2] dos Santos CC, Slutsky AS (2006). The contribution of biophysical lung injury to the development of biotrauma. Annu Rev Physiol.

[3] Razazi K, Thille AW, Carteaux G, Beji O, Brun-Buisson C, Brochard L, Mekontso Dessap A (2014). Effects of pleural effusion drainage on oxygenation, respiratory mechanics, and hemodynamics in mechanically ventilated patients. Ann Am Thorac Soc.

[4] Goligher EC, Leis JA, Fowler RA, Pinto R, Adhikari NK, Ferguson ND (2011). Utility and safety of draining pleural effusions in mechanically ventilated patients: a systematic review and meta-analysis. Crit Care.

[5] Marie C, Losser MR, Fitting C, Kermarrec N, Payen D, Cavaillon JM (1997). Cytokines and soluble cytokine receptors in pleural effusions from septic and nonseptic patients. Am J Respir Crit Care Med.

[6] Davies LC, Jenkins SJ, Allen JE, Taylor PR (2013). Tissue-resident macrophages. Nat Immunol.

[7] Liu M, Tanswell AK, Post M (1999). Mechanical force-induced signal transduction in lung cells. Am J Physiol.

[8] Wang N, Chretien J, Bignon J, Hirsch A (1985). The Pleura in Health and Disease.

[9] Waters CM, Glucksberg MR, Depaola N, Chang J, Grotberg JB (1996). Shear stress alters pleural mesothelial cell permeability in culture. J Appl Physiol (1985).

[10] Batra H, Antony VB (2015). Pleural mesothelial cells in pleural and lung diseases. J Thorac Dis.

[11] Waters CM, Chang JY, Glucksberg MR, DePaola N, Grotberg JB (1997). Mechanical forces alter growth factor release by pleural mesothelial cells. Am J Physiol.

[12] Baldi RF KM, Thomas C, Sabbat T, Wang D, Tsatsari S, Young K, Soni S, O’Dea KP, Takata M, Wilson MR (2023). Ventilator-induced lung injury promotes inflammation within the pleural cavity in a mouse model. Am J Respir Crit Care Med.

[13] Wilson MR, Petrie JE, Shaw MW, Hu C, Oakley CM, Woods SJ, Patel BV, O’Dea KP, Takata M (2017). High-Fat Feeding Protects Mice From Ventilator-Induced Lung Injury, Via Neutrophil-Independent Mechanisms. Crit Care Med.

[14] Koh MW, Baldi RF, Soni S, Handslip R, Tan YY, O’Dea KP, Malesevic M, McAuley DF, O’Kane CM, Patel BV, Takata M (2021). Secreted Extracellular Cyclophilin A Is a Novel Mediator of Ventilator-induced Lung Injury. Am J Respir Crit Care Med.

[15] Wilson MR, Wakabayashi K, Bertok S, Oakley CM, Patel BV, O’Dea KP, Cordy JC, Morley PJ, Bayliffe AI, Takata M (2017). Inhibition of TNF Receptor p55 By a Domain Antibody Attenuates the Initial Phase of Acid-Induced Lung Injury in Mice. Front Immunol.

[16] Soni S, O’Dea KP, Tan YY, Cho K, Abe E, Romano R, Cui J, Ma D, Sarathchandra P, Wilson MR, Takata M (2019). ATP redirects cytokine trafficking and promotes novel membrane TNF signaling via microvesicles. FASEB J.

[17] Oakley C, Koh M, Baldi R, Soni S, O’Dea K, Takata M, Wilson M (2019). Ventilation following established ARDS: a preclinical model framework to improve predictive power. Thorax.

[18] Cailhier JF, Sawatzky DA, Kipari T, Houlberg K, Walbaum D, Watson S, Lang RA, Clay S, Kluth D, Savill J, Hughes J (2006). Resident pleural macrophages are key orchestrators of neutrophil recruitment in pleural inflammation. Am J Respir Crit Care Med.

[19] Ghosn EE, Cassado AA, Govoni GR, Fukuhara T, Yang Y, Monack DM, Bortoluci KR, Almeida SR, Herzenberg LA, Herzenberg LA (2010). Two physically, functionally, and developmentally distinct peritoneal macrophage subsets. Proc Natl Acad Sci U S A.

[20] Kim KW, Williams JW, Wang YT, Ivanov S, Gilfillan S, Colonna M, Virgin HW, Gautier EL, Randolph GJ (2016). MHC II+ resident peritoneal and pleural macrophages rely on IRF4 for development from circulating monocytes. J Exp Med.

[21] Chow A, Schad S, Green MD, Hellmann MD, Allaj V, Ceglia N, Zago G, Shah NS, Sharma SK, Mattar M, Chan J (2021). Tim-4(+) cavity-resident macrophages impair anti-tumor CD8(+) T cell immunity. Cancer Cell.

[22] Kuiper JW, Groeneveld AB, Slutsky AS, Plotz FB (2005). Mechanical ventilation and acute renal failure. Crit Care Med.

[23] Plotz FB, Slutsky AS, van Vught AJ, Heijnen CJ (2004). Ventilator-induced lung injury and multiple system organ failure: a critical review of facts and hypotheses. Intensive Care Med.

[24] Wakabayashi K, Wilson MR, Tatham KC, O’Dea KP, Takata M (2014). Volutrauma, but not atelectrauma, induces systemic cytokine production by lung-marginated monocytes. Crit Care Med.

[25] Stathopoulos GT, Zhu Z, Everhart MB, Kalomenidis I, Lawson WE, Bilaceroglu S, Peterson TE, Mitchell D, Yull FE, Light RW, Blackwell TS (2006). Nuclear factor-kappaB affects tumor progression in a mouse model of malignant pleural effusion. Am J Respir Cell Mol Biol.

[26] Dorr AD, Wilson MR, Wakabayashi K, Waite AC, Patel BV, van Rooijen N, O’Dea KP, Takata M (2011). Sources of alveolar soluble TNF receptors during acute lung injury of different etiologies. J Appl Physiol (1985).

[27] Porcel JM, Light RW (2006). Diagnostic approach to pleural effusion in adults. Am Fam Physician.

[28] Wiesolek HL, Bui TM, Lee JJ, Dalal P, Finkielsztein A, Batra A, Thorp EB, Sumagin R (2020). Intercellular Adhesion Molecule 1 Functions as an Efferocytosis Receptor in Inflammatory Macrophages. Am J Pathol.

[29] Soni S, O’Dea KP, Abe E, Khamdan M, Shah SV, Sarathchandra P, Wilson MR, Takata M (2022). Microvesicle-Mediated Communication Within the Alveolar Space: Mechanisms of Uptake by Epithelial Cells and Alveolar Macrophages. Front Immunol.

[30] Ordonez NG (2005). D2-40 and podoplanin are highly specific and sensitive immunohistochemical markers of epithelioid malignant mesothelioma. Hum Pathol.

[31] Grygorczyk R, Furuya K, Sokabe M (2013). Imaging and characterization of stretch-induced ATP release from alveolar A549 cells. J Physiol.

[32] Takahara N, Ito S, Furuya K, Naruse K, Aso H, Kondo M, Sokabe M, Hasegawa Y (2014). Real-time imaging of ATP release induced by mechanical stretch in human airway smooth muscle cells. Am J Respir Cell Mol Biol.

[33] Antony VB (2003). Immunological mechanisms in pleural disease. Eur Respir J.

[34] Matusali G, Trionfetti F, Bordoni V, Nardacci R, Falasca L, Colombo D, Terri M, Montaldo C, Castilletti C, Mariotti D, Del Nonno F (2021). Pleural Mesothelial Cells Modulate the Inflammatory/Profibrotic Response During SARS-CoV-2 Infection. Front Mol Biosci.

[35] Agostoni E, Zocchi L (2007). Pleural liquid and its exchanges. Respir Physiol Neurobiol.

[36] Pearse DB, Searcy RM, Mitzner W, Permutt S, Sylvester JT (2005). Effects of tidal volume and respiratory frequency on lung lymph flow. J Appl Physiol (1985).

[37] Cohen TS, Cavanaugh KJ, Margulies SS (2008). Frequency and peak stretch magnitude affect alveolar epithelial permeability. Eur Respir J.

