# Supplementary figures and images for: Ventilator-induced lung injury promotes inflammation within the pleural cavity
Source: Am J Respir Cell Mol Biol. Author manuscript; Available in PMC 2024 Jul 7. (PMC11225872; doi:10.1165/rcmb.2023-0332OC)

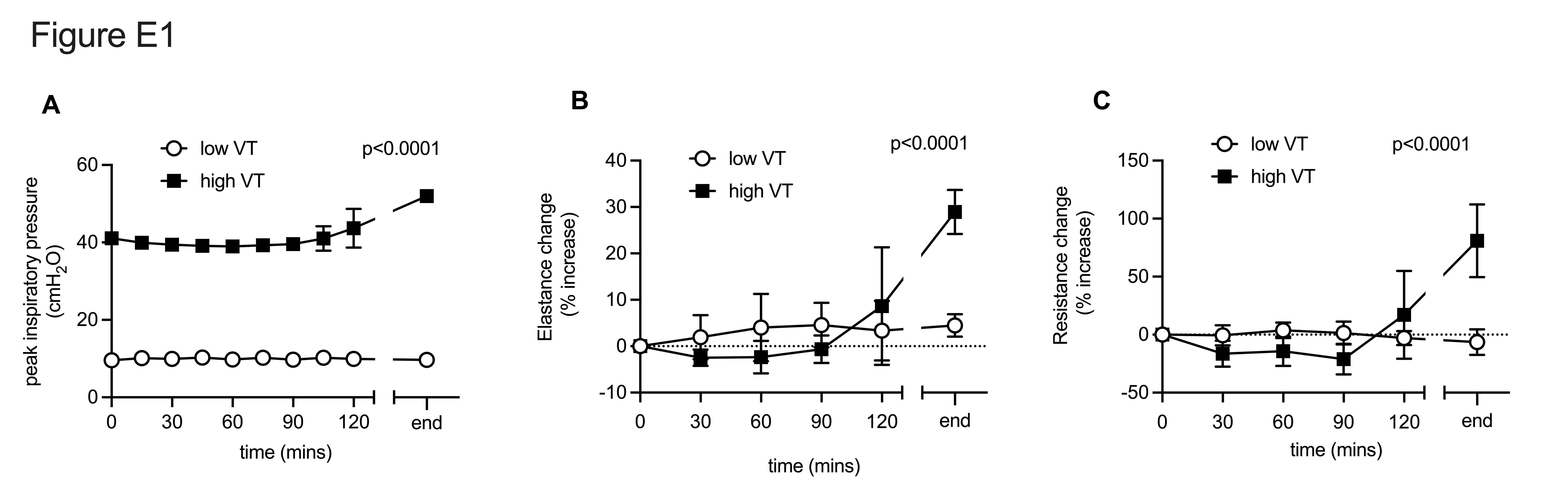

Supplement: Fig. E1 [file EMS196330-supplement-Fig__E1.tiff]

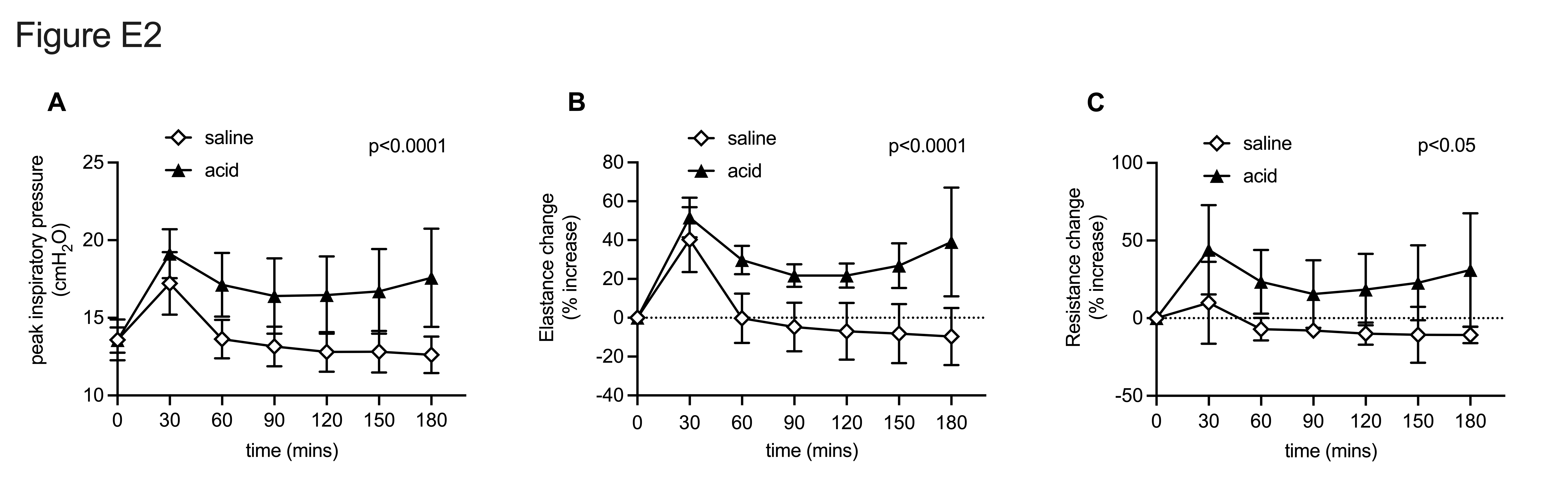

Supplement: Fig. E2 [file EMS196330-supplement-Fig__E2.tiff]

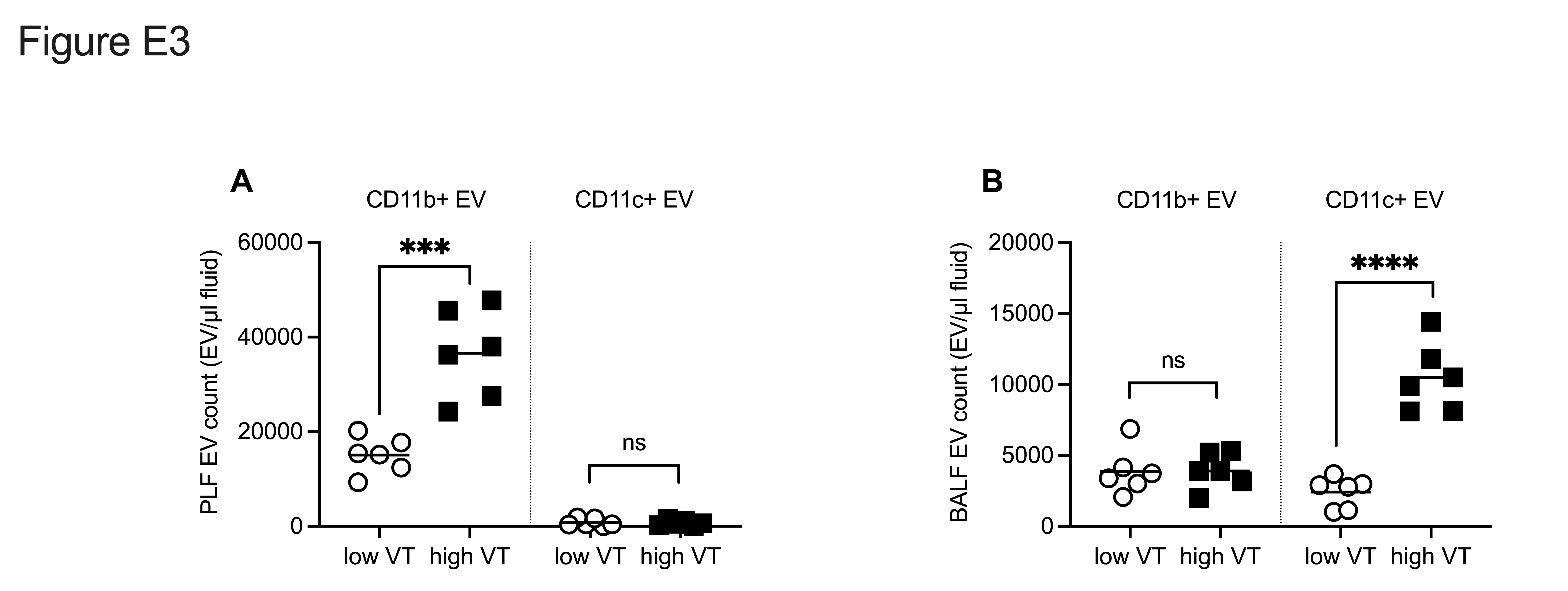

Supplement: Fig. E3 [file EMS196330-supplement-Fig__E3.tiff]

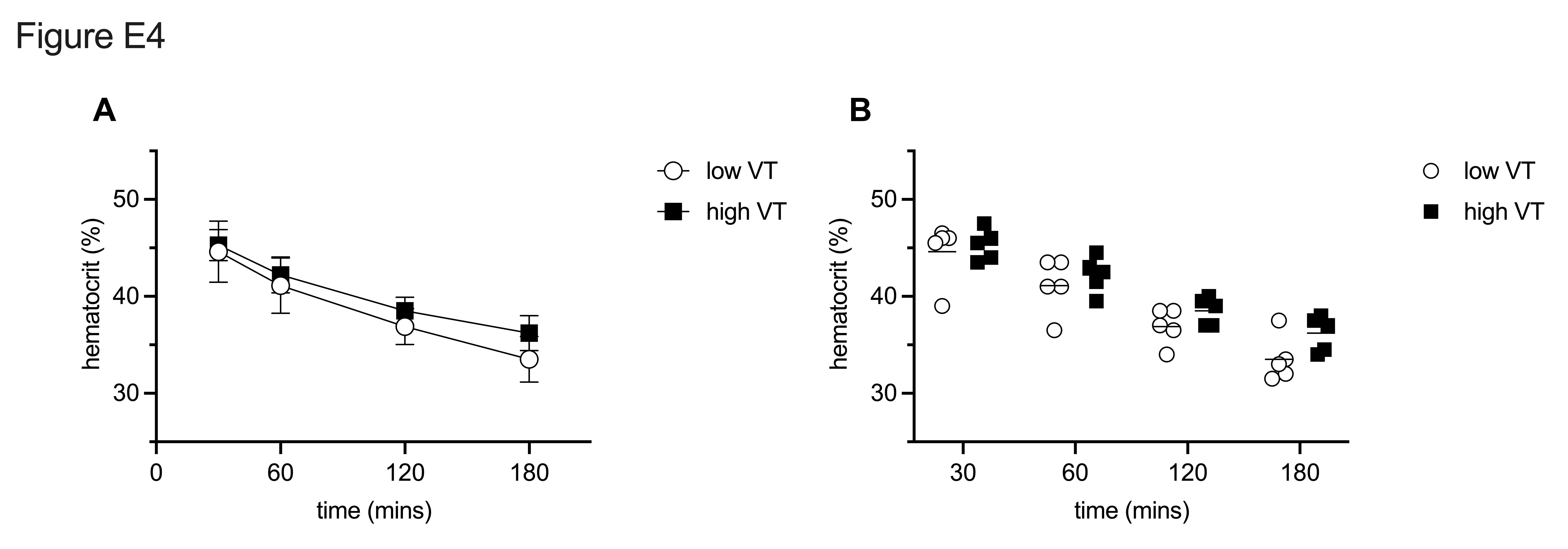

Supplement: Fig. E4 [file EMS196330-supplement-Fig__E4.tiff]

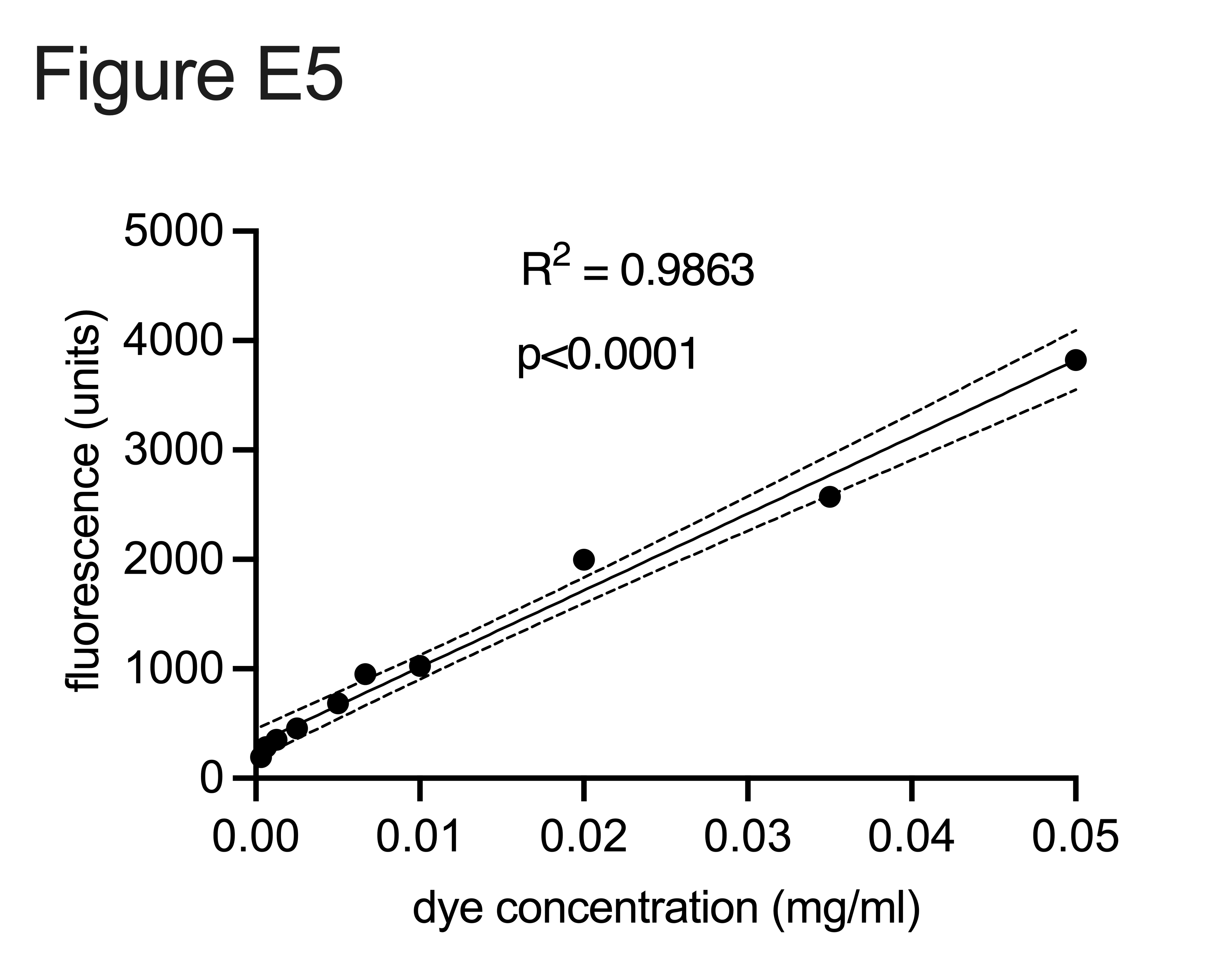

Supplement: Fig. E5 [file EMS196330-supplement-Fig__E5.tiff]
