# Supplementary material for: Ventilator-induced lung injury promotes inflammation within the pleural cavity
Source: Am J Respir Cell Mol Biol. Author manuscript; Available in PMC 2024 Jul 7. (PMC11225872; doi:10.1165/rcmb.2023-0332OC)
Supplement: Supplement [file EMS196330-supplement-Supplement.docx]

**Ventilator-induced lung injury promotes inflammation within the pleural cavity**

Rhianna F Baldi, Marissa W Koh, Chubicka Thomas, Tomasz Sabbat, Bincheng Wang, Stefania Tsatsari, Kieron Young, Alexander Wilson-Slomkowski, Sanooj Soni, Kieran P O’Dea, Brijesh V Patel, Masao Takata and Michael R Wilson

**Online supplement**

**Supplemental methods**

**Ventilator-induced lung injury model**

Male C57BL/6 mice (Charles River) aged 8-12 weeks (body weight 24-29g) were used throughout. Animal experiments were carried out under the Animals (Scientific Procedures) Act 1986, UK, with approval of the Imperial College London ethical review board. The experimental ventilatory protocol has been described in detail previously (E1-E3). In brief, mice were anesthetised with 80mg/kg ketamine and 8mg/kg xylazine and surgically prepared for cannulation of the left carotid artery to enable blood pressure measurement and fluid infusion (0.4ml/hr of 10U/ml heparin:saline). Mice were tracheostomised, connected to a custom-made ventilator-pulmonary function testing system (E1), and ventilated using a protective low tidal volume strategy (7-8ml/kg tidal volume (V_T_), 3cmH_2_O positive end-expiratory pressure (PEEP), 120 breaths per minute respiratory rate, using 100% O_2_). Following baseline measurements, mice were randomly allocated either to continue on the low V_T_ strategy, or switched to injurious high V_T_ ventilation (V_T_ 32-36ml/kg, 3cmH_2_O PEEP, 80 breaths per minute respiratory rate, using 96% O_2_/4%CO_2_). Mice ventilated with low V_T_ only, received a sustained inflation (30cmH_2_O, 5 seconds) every 30 minutes to minimise derecruitment. Mice were ventilated for pre-determined periods of up to a maximum of 3 hours, or until a mortality surrogate (peak inspiratory pressure increase >25% and/or blood pressure <50mmHg) was reached if this occurred earlier, in line with our previous studies (E1-E3).

For pharmacological inhibition of purinergic receptor signaling, a 50μl bolus of 50mM pyridoxalphosphate-6-azophenyl-2',4'-disulfonic acid (PPADS) tetrasodium (Tocris, Biotechne), a non-specific P2 purinergic receptor antagonist, or equal volume of vehicle (saline) was delivered intrapleurally immediately before the initiation of 1 hour high or low V_T_ ventilation. For intrapleural delivery, mice were attached to the ventilator but exposed to continuous positive airway pressure. The instilled solution was placed into the hub of a 23G needle which was then slowly advanced through the intercostal muscles. The fluid was drawn into the pleural cavity when the needle tip penetrated into the sub-atmospheric pressure pleural space, once mice began to recover spontaneous breathing.

**Acid-induced lung injury model**

As a comparison to the VILI model, experiments using an acute acid aspiration lung injury model were carried out, as described in detail previously (E4-E5). In brief, mice were anesthetised and instrumented as described above, and ventilated using non-injurious low V_T_ settings. Following baseline measurements, mice were intratracheally instilled with 50µl of either saline or 0.15M HCl. Four recruitment manoeuvres were carried out to disperse the fluid bolus, and animals were ventilated with the same low V_T_ settings for a further 3 hours, with recruitment manoeuvres every 30 minutes. (Note that unlike the VILI model, all animals were ventilated for the full 3 hour period). Mice were then terminated and samples collected as described.

**Mediator measurements**

Mice were terminated by exsanguination and blood samples were collected. Additionally, bronchoalveolar lavage fluid (BALF) was collected by instilling 750µl saline into the lungs via the endotracheal tube and withdrawing as much as possible without producing substantial negative pressure. Pleural lavage fluid (PLF) was collected by piercing the parietal pleura of the right lung between the 4^th^ and 5^th^ ribs, and introducing a fine cannula into the pleural cavity. 400µl saline was then instilled, and as much fluid as possible recovered. BALF and PLF samples were centrifuged (300g, 5 minutes, 4ºC) to produce cell-free supernatants. Blood was centrifuged at 1500g (5 minutes, 4ºC) to produce plasma samples.

Concentration of inflammatory mediators CXCL1, CCL2, IL-6 and IL-1β were determined in samples by ELISA according to manufacturers’ instructions (RnD Systems, Biotechne). Total protein concentration was evaluated using Bio-Rad protein assay reagent (Bio-Rad). In experiments to determine ATP concentration in PLF, the pleural lavage procedure was carried out using 20mM ARL67156 (ecto-ATPase inhibitor) in 400µl saline to minimise degradation of ATP. Cell free supernatant or ATP standard were incubated with substrate solution (ATPlite, PerkinElmer) in a 96 well plate with shaking, and luminescence determined (FLx800, BioTek Instruments).

**Protein movement studies**

In some VILI experiments the movement of protein (albumin) into or out of the pleural cavity and alveolar space was determined by introduction of fluorescent Alexa-Fluor 594-conjugated albumin (ThermoFisher Scientific).

*Blood to pleural cavity / alveolar space:*

To determine blood-to-pleural cavity and blood-to-alveolar space permeability, a 50µl bolus of 5mg/ml AF594-albumin was injected intravenously into the circulation at the start of ventilation (E6). After 3 hours low or high V_T_ ventilation samples of plasma, BALF and PLF were collected as described above, and fluorescence in cell-free supernatants determined as described below. Plasma fluorescence was determined after 100-fold dilution (in saline) while BALF and PLF samples were measured without dilution. Dye movement was evaluated as the ratio of fluorescence units in BALF or PLF versus fluorescence units in plasma (x100) (E6).

*Pleural cavity to blood movement:*

To determine pleural cavity-to-blood movement of protein, 50µl 5mg/ml AF594-albumin was instilled into the pleural cavity of mice using the technique describe above for pharmacological inhibition. Following administration, mice were returned to the ventilator and randomised to receive either high or low V_T_ ventilation. Note that in order to ensure that all animals were exposed to the dye for the same length of time, high V_T_ settings were somewhat reduced (27-33ml/kg) compared to those in other experiments (i.e. to ensure that no animals met the mortality surrogate early). Serial 70µl samples of blood were collected from the carotid artery cannula at intervals throughout (30 min, 1 hour, 2 hours and 3 hours) and separated in a hematocrit centrifuge, and fluorescence determined in the plasma fraction.

*Alveolar space to blood movement:*

To determine alveolar space-to-plasma movement of protein, mice were detached temporarily from the ventilator and 50µl AF594-albumin was instilled intratracheally via the endotracheal tube. Repeated (x4) sustained inflation manoeuvres (5 seconds, 30cmH_2_O) were carried out to disperse fluid. In order to mitigate the acute impacts of intratracheal fluid administration on respiratory mechanics as far as possible, mice were ventilated with low V_T_ settings for 15 minutes followed by a final recruitment manoeuvre before being randomised to receive either high or low V_T_. To make the high V_T_ ventilation as comparable as possible between intrapleurally and intratracheally administered mice (despite lingering effects of intratracheal administration on mechanics) we chose to ventilate high V_T_ animals with a matched plateau pressure (34.3±2.27 vs 33.3±1.47 cmH_2_O for intrapleural dye vs intratracheal dye respectively; N=6). For non-injurious ventilation effects of route of dye administration on mechanics were ignored and all animals were ventilated with V_T_ 7-8 ml/kg. Serial 70µl samples of blood were collected from the carotid artery cannula at intervals throughout (30 min, 1 hour, 2 hours and 3 hours) and separated in a hematocrit centrifuge, and fluorescence determined in the plasma fraction.

For both pleural cavity-to-blood and alveolar space-to-blood measurements, plasma fluorescence was determined in undiluted samples, while initial instillate was diluted 100-fold before measurement. Dye movement was evaluated as the ratio of fluorescence units in plasma versus fluorescence units in the initial instillate (x100).

Within our experimental set-up, all animals receive a constant fluid infusion of 0.9% saline with 10 U/ml heparin, at a rate of 0.4ml/hour, to compensate for insensible fluid loss mainly via exhaled breath. Due to the lower minute volume of low V_T_ animals compared to high V_T_, it is possible that these animals may have a slight relative hemodilution. To consider this possibility, we determined the hematocrit within low and high V_T_ animals, and showed that while this decreased somewhat throughout ventilation, this was no different between strategies (Fig E4). Thus we have confidence that differences between ventilation strategies are a result of enhanced movement of protein dye, rather than any dilution effects.

*Fluorescence measurements:*

Cell-free samples of pleural fluid, BAL or plasma, or samples of initially instilled dye, were analysed (with appropriate dilutions as described above) for AF-594 fluorescence using a fluorescent plate reader (FLx800, Biotek Instruments).

For fluorescence measurements, samples of 10μl volume were placed in triplicate into black 384-well plates and centrifuged at 200g for 1 minute to ensure samples filled the bottom of the well. Plates were then read using filters set at 590/20 nm excitation and 635/32 nm emission wavelengths, with x50 sensitivity. Fluorescence readouts were linear over the range of dilutions used (Fig E5).

**Flow cytometry**

Flow cytometry was used to explore the populations of cells within the pleural cavity that may contribute to inflammatory responses, focusing primarily on resident macrophages. PLF samples were collected and stained with antibodies against CD11b (clone M1/70; PE/Dazzle^TM^ 594), F480 (clone BM8; APC), MHCII (clone M5/114.15.2; PerCP), ICAM2 (clone 3C4 (MIC2/4); FITC), TIM-4 (clone RMT4-54; PE), CD19 (clone 1D3/CD19; FITC or PE), CD86 (clone GL1; PE), Ly6G (clone 1A8; APC Cyanine 7) and Ly6C (clone HK1.4; PE Cyanine 7) before addition of counting beads for quantification in a CyanADP Flow cytometer (Beckman Coulter). In addition, in some experiments cells were stained for surface expression of ICAM-1 (clone YN1/1.7.4; FITC) or permeabilised and stained for IL-1β (clone NJTEN3; PE) expression as markers of cell activation.

Remaining unstained PLF samples were centrifuged to remove cells (300g, 5 minutes, 4ºC) leaving extracellular vesicles within the cell-free supernatant. These were stained with antibodies against CD11b, CD11c (clone N418; APC Cyanine 7), CD19 and T1α (clone 8.1.1; PE Cyanine 7) and quantified by flow cytometry as described by us previously (E7, E8). Samples from in vitro stretch and in vivo inhibition studies were analysed using a Cytek NL-3000 flow cytometer (Cytekbio).

All antibodies were purchased from Biolegend except for PE-CD86 and PE-IL-1β which were purchased from eBioscience.

**In vitro stretch studies**

4/4 R.M.-4 rat pleural mesothelial cells (ATCC) were cultured in complete F12K Medium (ATCC) containing 15% foetal calf serum and 5% penicillin-streptomycin until confluent. Cells were then seeded onto collagen-coated flexible bottomed 6-well plates (BioFlex®, Flexcell International) and exposed to a maximum of 20% cyclic mechanical stretch (0.25 Hz, duty cycle 33.3%) using a Flexcell® FX-6000™ Tension System. Static controls were seeded into the same culture plates with rubber bungs placed underneath wells to prevent stretching. Pleural mesothelial cells were exposed to stretch or remained static for 15 minutes for measurement of ATP release or 4 hours for measurement of EV release.

After 15 minutes, supernatants were collected and incubated with ARL67156 (ecto-ATPase inhibitor; Tocris), and ATP concentration was measured as described above. Alternatively, after 4 hours of stretch, supernatants were harvested and centrifuged at 300g for 5 minutes at 4°C to obtain cell-free supernatant. This was stained with an antibody against the pleural mesothelial marker T1α (clone PMab-2; AlexaFluor®647) and EVs were quantified using flow cytometry. Supernatants were also evaluated for lactate dehydrogenase (LDH) levels using LDH-Cytox Assay kit (Biolegend) according to manufacturer’s instructions.

**Statistics**

All data were evaluated for normal distribution by Shapiro-Wilk test of residuals. Normally distributed data were analysed by t-test or one-way ANOVA with Sidak’s test for end-point samples, or by two-way ANOVA with Tukey’s multiple comparisons test for time-course studies. Non-normally distributed data were analysed by Mann-Whitney test or by Kruskal-Wallis followed by Dunn’s test. Time course studies are displayed as mean ± standard deviation or individual data points, while end-point studies are displayed as individual data points with bars showing either mean or median values.

**REFERENCES**

E1. Wilson MR, Patel BV, Takata M. Ventilation with "clinically relevant" high tidal volumes does not promote stretch-induced injury in the lungs of healthy mice. *Crit Care Med* 2012; 40: 2850-2857

E2. Wilson MR, Petrie JE, Shaw MW, Hu C, Oakley CM, Woods SJ, Patel BV, O'Dea KP, Takata M. High-Fat Feeding Protects Mice From Ventilator-Induced Lung Injury, Via Neutrophil-Independent Mechanisms. *Crit Care Med* 2017; 45: e831-e839.

E3. Koh MW, Baldi RF, Soni S, Handslip R, Tan YY, O'Dea KP, Malesevic M, McAuley DF, O'Kane CM, Patel BV, Takata M, Wilson MR. Secreted Extracellular Cyclophilin A Is a Novel Mediator of Ventilator-induced Lung Injury. *Am J Respir Crit Care Med* 2021; 204: 421-430

E4. Wilson MR, Wakabayashi K, Bertok S, Oakley CM, Patel BV, O'Dea KP, Cordy JC, Morley PJ, Bayliffe AI, Takata M. Inhibition of TNF Receptor p55 By a Domain Antibody Attenuates the Initial Phase of Acid-Induced Lung Injury in Mice. *Front Immunol* 2017; 8: 128.

E5. Patel BV, Wilson MR, O'Dea KP, Takata M. TNF-induced death signaling triggers alveolar epithelial dysfunction in acute lung injury. *J Immunol* 2013; 190: 4274-4282

E6. Wilson MR, Goddard ME, O'Dea KP, Choudhury S, Takata M. Differential roles of p55 and p75 tumor necrosis factor receptors on stretch-induced pulmonary edema in mice. *Am J Physiol Lung Cell Mol Physiol* 2007; 293: L60-68

E7. Soni S, Wilson MR, O'Dea KP, Yoshida M, Katbeh U, Woods SJ, Takata M. Alveolar macrophage-derived microvesicles mediate acute lung injury. *Thorax* 2016; 71: 1020-1029

E8. Soni S, O'Dea KP, Tan YY, Cho K, Abe E, Romano R, Cui J, Ma D, Sarathchandra P, Wilson MR, Takata M. ATP redirects cytokine trafficking and promotes novel membrane TNF signaling via microvesicles. *FASEB J* 2019; 33: 6442-6455

**Supplemental data**

**Supplementary Figure E1. Time course of injury in VILI model.** Peak inspiratory pressure (A), elastance change (B) and resistance change (C) were measured across the course of experiments lasting either 3 hours or until mortality surrogate markers were met if that occurred sooner (high tidal volume (V_T_) animals started to meet mortality surrogates from 120 minutes onwards). Note that ‘end’ reflects the final measurement taken, regardless of the length of ventilation, and only 1 high V_T_ animal survived the entire 3 hour period. Data in B&C were calculated as percentage change in elastance or resistance compared to the value at the start of either high or low V_T_ ventilation. Data in all figures are displayed as mean±SD and evaluated by 2-way ANOVA. Significant differences (p<0.001) are shown for time x ventilation strategy interaction. N=6-7 for each dataset at each time point.

**Supplementary Figure E2. Time course of injury in acid aspiration model.** Peak inspiratory pressure (A), elastance change (B) and resistance change (C) were measured across the course of experiments lasting 3 hours. Data in B&C were calculated as percentage change in elastance or resistance compared to the value immediately following either saline or HCl instillation. Data in all figures are displayed as mean±SD and evaluated by 2-way ANOVA. Significant differences (p<0.05 or p<0.001) are shown for time x treatment interaction. N=6 for each dataset at each time point.

**Supplementary Figure E3. Quantification of macrophage extracellular vesicles in pleural lavage fluid (PLF) and bronchoalveolar lavage fluid (BALF) samples by flow cytometry.** Extracellular vesicles expressing surface markers for CD11b or CD11c were quantified in PLF (A) and BALF (B) after 1 hour of high or low V_T_ ventilation.  Data were normally distributed and analysed by t-tests.  Data are displayed as individual data points with solid line indicating mean value.  N=6 for each dataset.  ***p<0.001, ****p<0.0001.

**Supplementary Figure E4. Blood hematocrit during VILI.** During pleural fluid-to-blood protein movement studies, hematocrit values were determined after 30, 60, 120 and 180 minutes to clarify whether ventilation strategy induced differing degrees of hemodilution. Data were normally distributed and were analysed by 2-way ANOVA. Panel A shows time course of changes within different groups, with data displayed as mean ± SD. Time x group interaction showed no significant interaction so post-hoc analyses were not carried out. Panel B shows comparisons at each time point, displayed as individual data points. N=5 for each dataset at each time point.

**Supplementary Figure E5. Dye : fluorescence correlation**. In order to confirm that changes in fluorescence units measured during protein movement studies were linearly related to changes in concentration, AF594- albumin was diluted in saline to various concentrations and fluorescence determined. Panel shows mean fluorescence value of triplicates plotted against dye concentration, with 95% CI. Pearson correlation analysis showed R^2^ 0.9863 and p<0.0001 demonstrating a strong linear correlation over the concentrations of dye used and fluorescence units recorded in the study.
